# Endo-Lysosomal Dysfunction in Human Proximal Tubular Epithelial Cells Deficient for Lysosomal Cystine Transporter Cystinosin

**DOI:** 10.1371/journal.pone.0120998

**Published:** 2015-03-26

**Authors:** Ekaterina A. Ivanova, Maria Giovanna De Leo, Lambertus Van Den Heuvel, Anna Pastore, Henry Dijkman, Maria Antonietta De Matteis, Elena N. Levtchenko

**Affiliations:** 1 Department of Pediatric Nephrology & Growth and Regeneration, University Hospitals Leuven & Katholieke Universiteit, Leuven, Belgium; 2 Telethon Institute of Genetics and Medicine (TIGEM), Naples, Italy; 3 Department of Pediatric Nephrology, Radboud University Medical Center Nijmegen, Nijmegen, The Netherlands; 4 Laboratory of Proteomics and Metabolomics, Children‘s Hospital and Research Institute "Bambino Gesù" IRCCS, Rome, Italy; 5 Department of Pathology, Radboud University Nijmegen Medical Centre, Nijmegen, The Netherlands; Center for Molecular Biotechnology, ITALY

## Abstract

Nephropathic cystinosis is a lysosomal storage disorder caused by mutations in the *CTNS* gene encoding cystine transporter cystinosin that results in accumulation of amino acid cystine in the lysosomes throughout the body and especially affects kidneys. Early manifestations of the disease include renal Fanconi syndrome, a generalized proximal tubular dysfunction. Current therapy of cystinosis is based on cystine-lowering drug cysteamine that postpones the disease progression but offers no cure for the Fanconi syndrome. We studied the mechanisms of impaired reabsorption in human proximal tubular epithelial cells (PTEC) deficient for cystinosin and investigated the endo-lysosomal compartments of cystinosin-deficient PTEC by means of light and electron microscopy. We demonstrate that cystinosin-deficient cells had abnormal shape and distribution of the endo-lysosomal compartments and impaired endocytosis, with decreased surface expression of multiligand receptors and delayed lysosomal cargo processing. Treatment with cysteamine improved surface expression and lysosomal cargo processing but did not lead to a complete restoration and had no effect on the abnormal morphology of endo-lysosomal compartments. The obtained results improve our understanding of the mechanism of proximal tubular dysfunction in cystinosis and indicate that impaired protein reabsorption can, at least partially, be explained by abnormal trafficking of endosomal vesicles.

## Introduction

Nephropathic cystinosis (MIM 219800) is an autosomal recessive disorder caused by mutations in the *CTNS* gene encoding cystinosin, a lysosomal cystine transporter [1, 2]. Cystinosin is a 367-amino acid lysosomal membrane protein with predicted 7 transmembrane domains and two lysosomal targeting motifs located in the C-terminus and in the 5^th^ cytosolic loop [3]. A second isoform of cystinosin has been described, in which the C-terminal targeting motif is substituted by a longer sequence. This isoform has a different localization within the cell, being found on the plasma membrane, in the lysosomes and on other cytosolic structures such as endoplasmic reticulum and Golgi apparatus [4, 5]. Cystinosin was demonstrated to act as a proton-dependent transporter [6, 7], thus, effective cystine transport is strictly dependent on the acidic pH inside the lysosomal lumen. Cystinosin deficiency results in lysosomal cystine accumulation in all body organs and tissues. Treatment with cystine-lowering drug cysteamine forms the basis of current therapy of cystinosis [2, 8]. Cysteamine enters the lysosome and splits cystine molecule into cysteine and cysteine-cysteamine mixed disulphide. Both products can then be released from the lysosome through cysteine and PQLC2 transporters respectively [9].

The most severe infantile nephropathic clinical form of cystinosis is typically associated with mutations resulting in a complete loss of function of cystinosin [10]. Among the Caucasians originating from the Northern Europe, the most prevalent mutation is a 57-kb deletion, which affects the first 10 exons of the *CTNS* gene [1]. Cells deriving from patients bearing this deletion express no cystinosin and accumulate cystine in the lysosomes in normal culturing conditions [11].

The initial clinical manifestation of cystinosis developing during infancy is renal Fanconi syndrome, a generalized renal proximal tubular dysfunction, characterized by polyuria and abnormal urinary loss of amino acids, glucose, low-molecular-weight (LMW) and intermediate weight proteins and other solutes [2, 11, 12]. In humans, generalized aminoaciduria appears as the first biochemical sign, already in a neonatal period followed by glucosuria, phosphaturia and urinary loss of bicarbonate and proteins, gradually developing into a full-blown Fanconi syndrome by ~6 months of age [13]. Later in life, untreated patients develop progressive renal damage leading to the end-stage renal disease (ESRD) and multiple extra-renal complications affecting eyes, endocrine organs, liver, muscles and central nervous system [2, 14, 15]. Treatment with cysteamine prevents lysosomal accumulation of cystine, improves growth and postpones the progression of renal disease and the development extra-renal complications, however, it offers no cure for renal Fanconi syndrome, although some improvement has been reported in patients treated starting from the early age [14–18].

The mechanism of renal Fanconi syndrome in cystinosis is still not completely understood [11]. Impaired apical transporter function was demonstrated in cultured proximal tubular cells from cystinosis patients [19]. Reabsorption of proteins from the primary urine is performed by endocytosis and is dependent on the concerted functioning of multiligand scavenger receptors present on the apical surface of proximal tubules. Recent studies in cystinosis mouse model (Ctns^-/-^ mice) have shown a decreased expression of the multi-ligand receptors megalin and cubilin at the proximal tubule apical surfaces associated with cell dedifferentiation [20, 21]. Therefore, the impaired reabsorption in the affected proximal tubules can result either from the decreased expression of the multiligand receptors or from the impaired delivery of the receptors and, possibly, other transporters to the apical surface of proximal tubular cells due to deficient vesicular trafficking and recycling. A combination of both pathological changes is also possible. However, an accurate study of the endocytosis and vesicular trafficking in human proximal tubular cells deficient for cystinosin has not been performed.

The major challenge in studying the pathogenesis of renal dysfunction in cystinosis during many years was the absence of a suitable model. The first mouse model developed on a FVB/N genetic background showed no signs of kidney disease despite pronounced cystine accumulation in the kidney [22]. Subsequently, a novel murine model of cystinosis generated on a pure C57BL/6 background showed signs of proximal tubulopathy and kidney failure and therefore offered an opportunity for studying pathogenesis [23, 20, 21]. However, it remains unclear to which extent the pathogenic mechanisms described in mice can be extrapolated to human proximal tubular cells, especially taking into account that renal phenotype in mice do not fully replicate human disease [23]. Furthermore, it is not always conceivable to differentiate between pathologic and compensative mechanisms *in vivo* [21].

In our study we examined the direct consequences of cystinosin deficiency on proximal tubular reabsorption machinery in human proximal tubular cells after down-regulation of the *CTNS* gene with specific siRNA and confirmed the results in proximal tubular cells derived from patients with cystinosis. We found that the absence of cystinosin resulted in an acute disorganisation of endo-lysosomal compartments, with clustering of endocytotic vesicles in the perinuclear region, decreased expression of multiligand receptors on the cell surface and delayed processing of the ligands. These data, together with our finding of abnormal co-localization of late endosomal and lysosomal marker LAMP-1 with the motor protein kinesin-1 in cystinosin-deficient cells, suggest that cystinosin functions as an important regulator of endo-lysosomal dynamics. Thus, the absence of cystinosin might lead to a defective movement of endosomes to the cell surface. Importantly, pre-treatment with cysteamine resulted in partial improvement of the surface expression of receptors and rescued ligand processing, but could not restore the abnormal morphology of the endo-lysosomal compartments. Our work makes a contribution to the understanding of pathological processes leading to the renal Fanconi syndrome in cystinosis and provides further directions for studying possible treatments of the disease aimed to restore the defective trafficking mechanisms.

## Materials and Methods

### Antibodies and reagents

Human recombinant GST-RAP was produced and isolated as previously described [24]. Chemical reagents of analytical grade or higher were from Sigma-Aldrich. The antibodies used in this study include Alexa-488-conjugated anti-LAMP1 (Santa Cruz), anti-LAMP1 rabbit monoclonal antibody (Cell Signaling), anti-GST (16B12) (Covance), anti-EEA1 (Cell Signaling), anti-KIF5b (Millipore), anti-HA mouse monoclonal and rabbit polyclonal antibodies (Covance). The Alexa-Fluor-488, Alexa-Fluor-568 and Alexa-Fluor-633 secondary antibodies were from Life Technologies.

### Cell culture and transfection

Human kidney proximal tubular epithelial HK-2 cells originate from the American Tissue Type Collection (ATTC, USA) and were used to study the endocytosis previously [25]. Human conditionally immortalized PTEC (ciPTEC) were obtained from living kidney cells exfoliated into urine as described previously [26]. Proximal tubular cells were grown in DMEM-HAM’s F12 culture medium (Lonza) supplemented with 10% foetal bovine serum (Gibco), 50 IU/mL penicillin and 50 mg/mL streptomycin (Lonza). For ciPTEC, the medium was additionally supplemented with 5 μg/ml of insulin, 5 μg/ml of transferrin, 5 ng/ml of selenium, 40 pg/ml of tri-iodothyronine, 36 ng/ml of hydrocortisone and 10 ng/ml of EGF (all from Sigma) [26, 27]. HK-2 and ciPTEC cells were transfected with plasmid vectors using either TransIT-LT1 (Mirus Bio LLC) or jetPRIME (Polyplus Transfection), according to the manufacturer’s instructions, and incubated for 16–20 h before fixation.

### Down-regulation of the *CTNS* gene

The siRNA oligonucleotides targeting *CTNS* gene were purchased from Dharmacon (ON-TARGET plus SMART pool J-019914-05–08). HK-2 cells were transfected with siRNA using Dharmafect4 (Dharmacon) transfection reagent according to the manufacturer’s instruction and incubated for 2 to 3 days before performing further experiments. Down-regulation was confirmed by real-time RT-PCR using primers, specific for *CTNS*. Actin was used as a housekeeping gene control.

### Immunocytochemistry and confocal microscopy

For immunocytochemistry, cells grown on glass coverslips were fixed with 4% paraformaldehyde for 10 min at room temperature and washed once in PBS. Blocking reagent was added to the cells for 20 min, followed by a 1 h incubation with appropriate concentrations of the primary antibodies in blocking reagent (0.5% BSA, 0.05% saponine, 50 mM NH4Cl, 0.02% NaN3 in PBS). The cells were then washed with PBS and incubated with secondary antibodies (1:400) and DAPI (1:1000) for detection of nuclei, diluted in blocking solution. The cells were washed twice in PBS and once in ion-free water to remove salts. The coverslips were then mounted on glass microscope slides (Carlo Erba, Italy) with Mowiol (Calbiochem). The samples were examined under either Zeiss LSM 710 or Zeiss Axiovert 100M confocal laser-scanning microscopes, in both cases equipped with a 63X (NA 1.4) oil-immersion objective. 5–10 randomly chosen fields were analyzed for each condition. For RAP uptake experiments, at least 150 individual cells were analyzed for each time point. Image processing and analysis were performed using ImageJ software. Fluorescence intensity was measured in green and red channels after subtracting background inside a selected area corresponding to an individual cell. Microscopic images were prepared for publication using ImageJ (http://rsb.info.hin.gov/ij/) and Adobe Photoshop CS4 software for cropping. The measurement of the lysosomal size was performed as described previously [28]. Lysosome diameter was measured using ImageJ software, with lysosomes approximated as circles with the size from 0.1 to 5.0 μm^2^. Thresholds were kept the same for all images.

### Electron microscopy

For electron microscopy, small pieces of cystinotic kidney biopsy described previously [29] and control kidney biopsy available at the Pathology Department of Radboud University Medical Centre (Nijmegen, The Netherlands) obtained from a subject without cystinosis or cystinosis patient having Fanconi syndrome were fixed in 2.5% glutaraldehyde dissolved in 0.1 M sodium cacodylate buffer, pH 7.4 overnight at 4°C and washed in the same buffer. The tissue fragments were postfixed in palade-buffered 2% OsO_4_ for 1 h, dehydrated, and embedded in Epon 812 (Merck, Darmstadt, Germany). Ultrathin sections were cut on an ultratome (Leica, Reichert Ultracuts, Wien, Austria), and contrasted with 4% uranyl acetate for 45 min and subsequently with lead citrate for 4 min at room temperature. Sections were examined in a Jeol 1200 EX2 electron microscope (JEOL, Tokyo, Japan).

### Endocytosis assay

Cells were serum-starved for 1 h and washed twice in cold PBS containing 1% BSA. The cells were incubated for 30 min on ice with 2.5 μg/ml recombinant GST-RAP in serum-free medium. To measure surface binding of the ligand, cells were washed with cold complete medium and fixed in 4% paraformaldehyde. To assess the endocytosis, the cells were washed with complete medium and incubated at 37°C to allow internalization of the ligand at the indicated times. At the end of this incubation, the cells were briefly acid washed (150 mM NaCl, 10 mM acetic acid, pH 3.5) and fixed.

### Megalin uptake and recycling assay

For the HA-Meg4 internalisation assay control and CTNS KD HK-2 cells were transiently transfected with HA-Meg4 and pcDNA3-RAP, serum starved for 2 h at 37°C in serum-free DMEM, washed twice in cold PBS with 1% BSA, chilled to 4°C, and incubated with the anti-HA monoclonal antibody for indicated times in an ice/water bath, to label the cell surface. The unbound antibody was rinsed off with cold HEPES-buffered DMEM, and the cells were incubated in preheated complete medium for indicated times at 37°C. After internalisation, the antibodies remaining on the cell surface were removed by a 30 s acid wash (150 mM NaCl, 10 mM acetic acid, pH 3.5), with a brief wash in PBS, and then the cells were fixed.

### BSA degradation assay

Lysosomal degradation was measured as described previously [30]. Briefly, control and cystinosis ciPTEC grown in glass-bottom cell culture chambers (LabTek) were incubated with 5 μg/ml DQ-BSA and 5 μg/ml Alexa-Fluor-555-BSA (Life Technologies) at 37°C for 2 h, washed and analyzed under a confocal microscope. Cells treated with 100 nM of bafilomycin A1 were used as a control.

### Measurement of lysosomal pH

Control and cystinotic ciPTEC and control and CTNS KD HK-2 cells were loaded with 10 μM of acridine orange for 15 min at 37°C, washed in HEPES-buffered complete medium and analyzed under a confocal microscope. Acridine orange was excited at 450–490 nm and the signal recorded at 520–560 nm or >620 nm. Typically, 5–10 images were collected from 3 separate experiments and the red/green ratio was calculated.

## Results

### Morphology of endosomal compartments is altered in cystinosin-deficient cells

We studied the changes in the endo-lysosomal compartments in cystinosis using two working models. First, we used human proximal tubular HK-2 cell line treated with siRNA specific to the *CTNS* gene (CTNS KD). The efficacy of down-regulation was evaluated by real-time PCR prior to each experiment and was 80% or higher after 2 days. The cystine accumulation was not detectable after 2 days of down-regulation when the morphology and endocytosis assessments were performed. After a prolonged 4-day exposure, HK-2 CTNS KD cells demonstrated a ~7-fold (p<0.005) increase of cystine content in comparison to the control, confirming the disrupted function of the transporter. However, the formation of the dense cobblestone-like monolayer of HK-2 cells obstructed the imaging experiments at this time point. Next, we confirmed the results in ciPTEC that were established as described previously from living exfoliated cells extracted from urine of a cystinosis patient bearing the 57-kb deletion and of a healthy volunteer [19].

**Fig 1:**
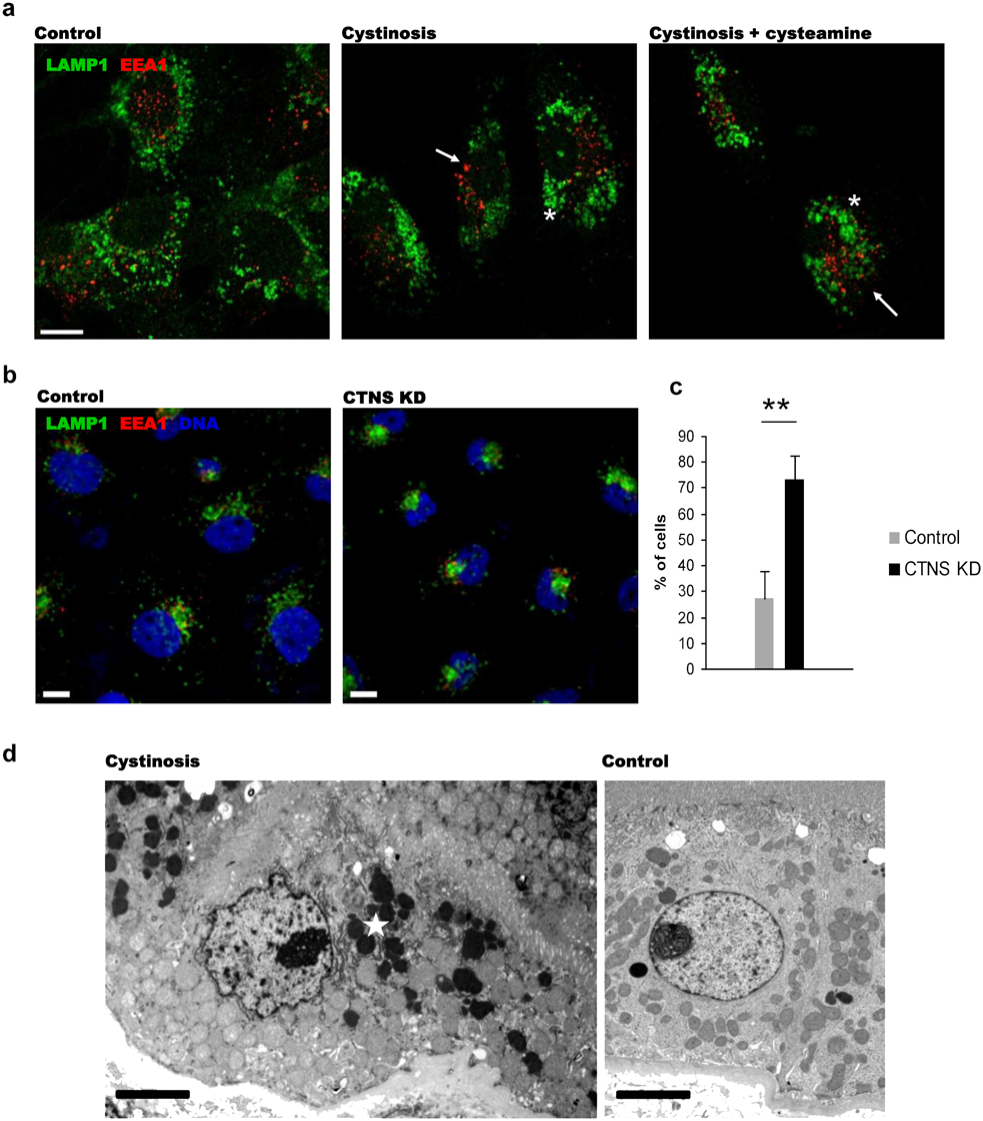
Altered endosomal/lysosomal compartments in cystinosin-deficient cells. (a) Altered morphology of early and late endosomes in ciPTEC deriving from the cystinosis patient: late endosomes appeared enlarged and abnormally clustered around the nuclei (*), and EEA1-positive endosomes tended to cluster in perinuclear area rather than to spread throughout the cell as in control ciPTEC (arrows). Cysteamine treatment had no influence on the abnormal phenotype in cystinotic cells. Scale bar = 10 μm. (b) Immunostaining with antibodies to early endosomal (EEA1) and late endosomal/lysosomal (LAMP1) markers revealed altered morphology in CTNS KD HK-2 cells. Scale bar = 10 μm. (c) Number of cells with “cystinotic” phenotype (endosomes clustered in perinuclear area and disappearance of peripheral vesicles) was quantified in CTNS KD cells; representation of 3 independent down-regulation experiments (** p<0.01). (d) Transmitted electron microscopy of kidney biopsy material obtained from a cystinosis patient treated with cysteamine and a control subject and. In cystinosis tissue, perinuclear localization of electron-dense lysosomes (asterisk) was demonstrated in PTEC. Scale bar = 3 μm.

To study the endosomal compartments, control and cystinosis ciPTEC cells and CTNS KD HK-2 cells were stained with antibodies specific to markers of early endosomes and late endosomes/lysosomes (EEA1 and LAMP1 respectively), the ER (VAP-A), Golgi (GM130) and TGN (TGN46). To measure the diameter of individual lysosomes, we used the method, described previously [28]. Clustered lysosomes were excluded from the analysis by the size filtering. In cystinosis ciPTEC, average size of the individual LAMP1-positive vesicles was enlarged by 35% (p<0.01), and the vesicles demonstrated a different distribution pattern than in the ciPTEC from a healthy donor (Fig. 1a). In HK-2 cells, accurate measurement of the lysosomal size was not feasible due to high level of clusterization of endosomal vesicles in the majority of CTNS KD cells: EEA1-positive and LAMP1-positive vesicles were abnormally clustered in perinuclear area (Fig. 1b). To estimate quantitatively the effect, two independent observers analyzed 2 distinct groups of cells, characterized by a ‘perinuclear’ localization of the vesicles, in which no labelled compartments were visible outside the compact perinuclear cluster, or by a ‘perinuclear+peripheral’ localization, in which endosomes were distributed throughout the central and peripheral areas. The former phenotype was defined as cystinotic, and the latter—as normal, since it was observed in most of the control HK-2 cells. In CTNS KD HK-2 cells, the number of cells with perinuclear distribution of endosomes increased by more than 2 folds in comparison with the control (Fig. 1c).

As cystinotic ciPTEC are characterized by a significant cystine accumulation under normal culture conditions, we tested whether cystine-lowering treatment could rescue the abnormal phenotype observed. Control and cystinosis ciPTEC cells were treated with 100 μM of cysteamine for 24 h. Such treatment was previously shown to decrease the cystine accumulation by ~91% without causing toxic effects on cultured cells. Longer treatment did not result in a significant further decrease of cystine content (S1 Fig.). We found that the cysteamine treatment had no significant effect on lysosomal size and distribution (Fig. 1a).

To confirm our *in vitro* findings, we performed electron microscopy study of a biopsy material obtained from a cystinosis patient treated with cysteamine that was previously described [29]. We found an increased number of electron-dense lysosomes clustered in the perinuclear region in proximal tubular cells compared to control tissue samples (Fig. 1d).

To identify different endosomal compartments, we transfected control and CTNS KD HK-2 cells with GFP-tagged Rab GTPases, commonly used for this purpose [31–33]. The co-localization of Rab5 and Rab7, corresponding to early and late endosomes, with EEA1 and LAMP1 markers respectively, was unchanged, and no mixing of the different endocytic compartments was detected in CTNS KD cells. The subcellular localization of Rab6 and Rab9, corresponding to trans-Golgi network and late endosome-to-Golgi transport domains, was also unaffected.

### Distribution of kinesin-1 is altered in cystinosin-deficient cells

**Fig 2:**
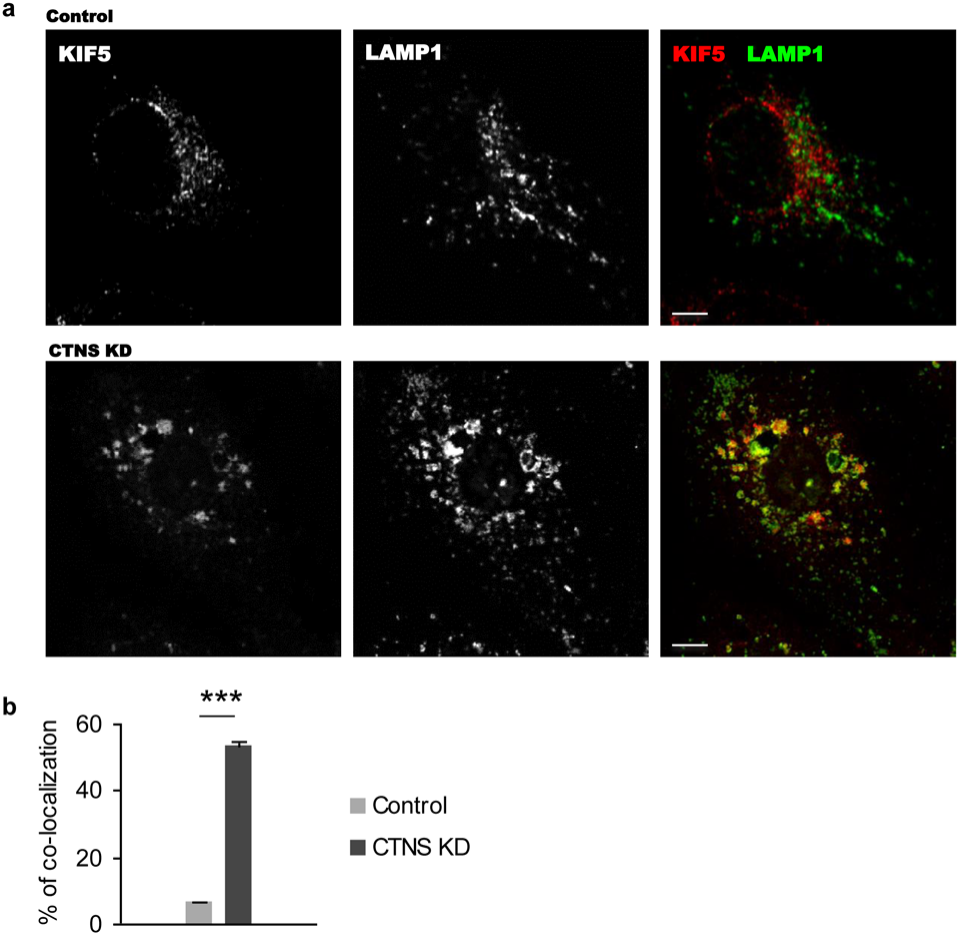
Abnormal distribution of kinesin-1 motor protein in cystinosin-deficient cells. (a) Control and CTNS KD HK-2 cells were fixed and stained with antibodies specific to KIF5b, a component of kinesin-1 complex, and LAMP1. CTNS KD cells had an abnormal distribution of kinesin-1 to LAMP1-positive enlarged structures. Scale bar = 10 μm. (b) Co-localization analysis was performed using ImageJ JACoP plug-in on 50 control and CTNS KD cells. CTNS KD cells had a 8-fold increase of co-localization between KIF5b and LAMP1 (*** p<0.001).

The perinuclear clustering of lysosomes suggested a possible involvement of the microtubule-based motor protein kinesin-1 responsible for the transport of endosomal vesicles towards microtubules plus-end. Visualization of kinesin-1 with anti-kinesin-1 heavy chain B (KIF5b) antibodies showed an altered subcellular localization of the protein in CTNS KD HK-2 cells as compared to control cells. Moreover, a ~8-fold increase of co-localization of kinesin-1 with LAMP1 was observed (Fig. 2a, b, p<0.001). Together these observations suggest that the altered endosomal and lysosomal trafficking and distribution in cystinosin-deficient cells might be explained by incorrect dynamics of lysosome-associated motor proteins, as discussed later.

### Multiligand receptor-mediated endocytosis is impaired in cystinosin-deficient cells

**Fig 3:**
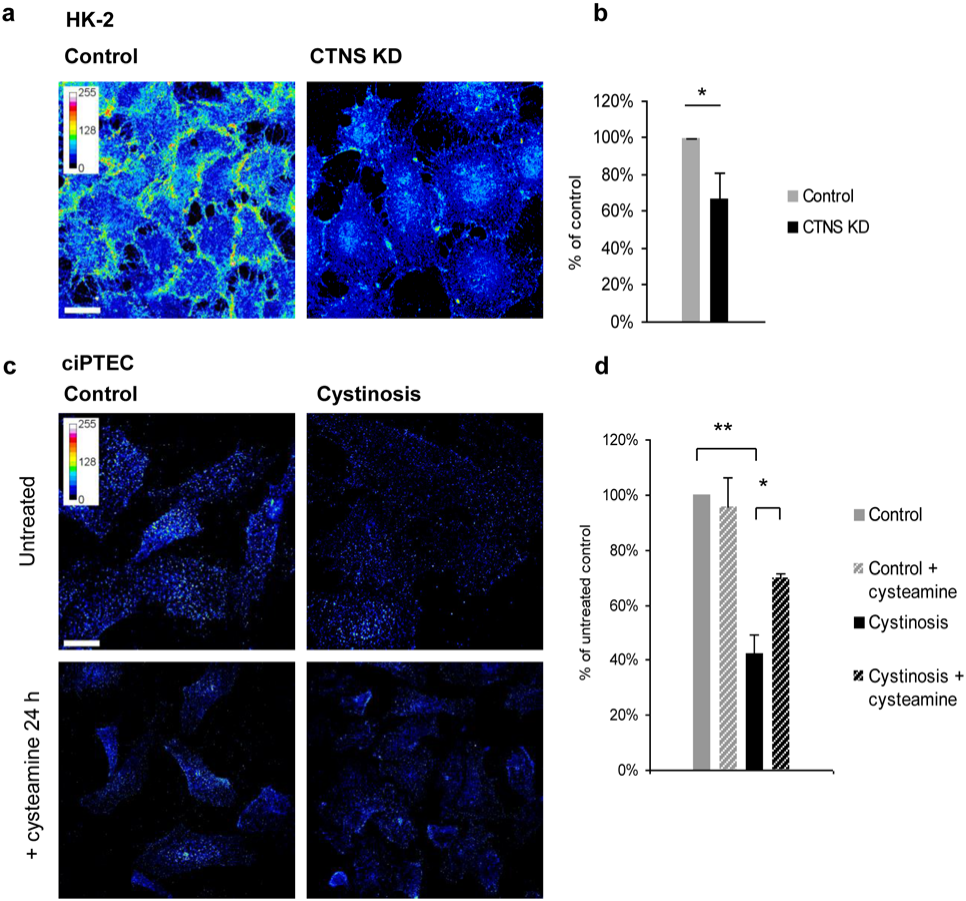
Decreased surface expression of megalin in cystinosin-deficient cells. (a, b) Control and CTNS KD HK-2 cells were incubated with GST-RAP for 30 min on ice. As evaluated by fluorescence intensity of individual cells, surface expression of multiligand receptors was decreased in cystinosin-deficient cells (* p<0.05). (c, d) ciPTEC cells from healthy donor and cystinosis patient were incubated with GST-RAP. Cystinotic cells demonstrated a decreased surface expression of multiligand receptors (** p<0.01). Treatment of cystinotic and control ciPTEC with 100 μM cysteamine for 24 h partially restored GST-RAP surface binding (* p<0.05). Scale bar = 10 μm.

To estimate the surface expression of megalin, we used a recombinant GST-tagged RAP protein, which binds megalin with high affinity and has been used to study endocytosis in proximal tubular cells previously [25]. HK-2 or ciPTEC cells were incubated with exogenous GST-RAP at 4°C for 30 minutes, fixed and stained with antibodies against GST to visualise surface-bound GST-RAP. We found that surface binding of GST-RAP was reduced by ~30% (p<0.05) in CTNS KD HK-2 cells (Fig. 3a, b) and by ~60% (p<0.01) in ciPTEC derived from a cystinosis patient (Fig. 3c, d) as compared to control. These observations indicate a reduced surface expression of megalin in cells deficient for cystinosin. Treatment of cystinotic ciPTEC with 100 μM of cystine-lowering drug cysteamine for 24 h resulted in a partial improvement of GST-RAP surface binding (Fig. 3c, d).

**Fig 4:**
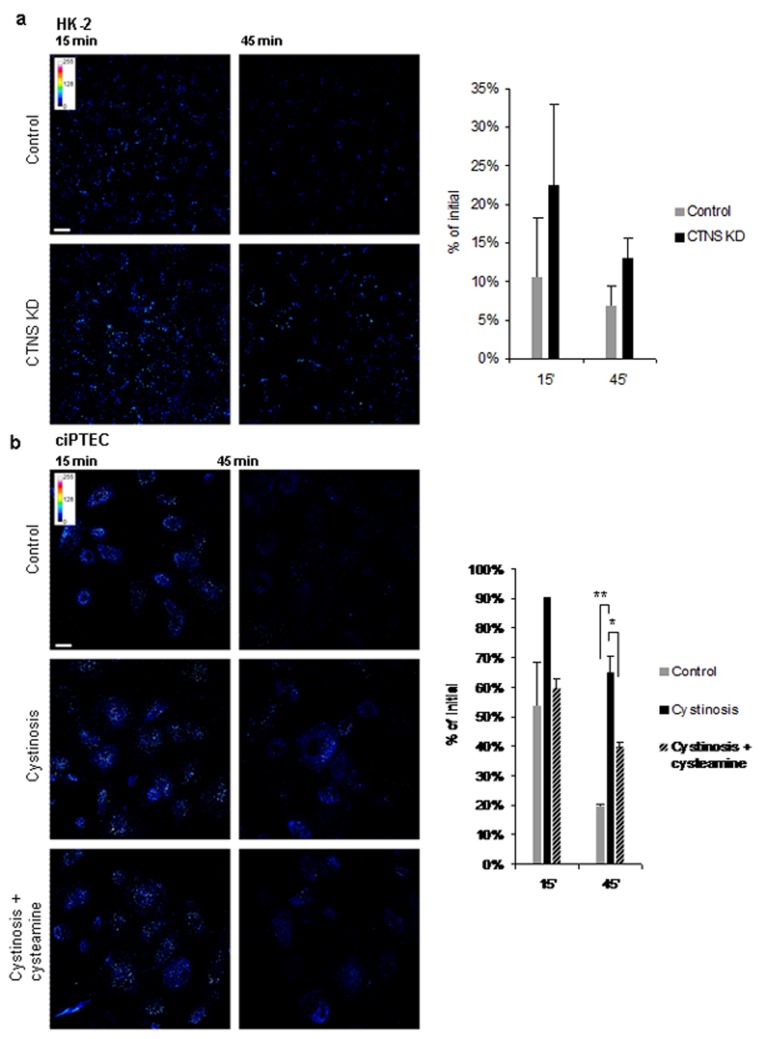
Endocytic uptake and processing of GST-RAP ligand. (a) Control and CTNS KD HK-2 cells were incubated with GST-RAP for 30 min on ice and then were allowed to internalize the ligand at 37°C for 15 and 45 min. GST-RAP processing was evaluated by the intensity of anti-GST staining with specific antibodies at indicated time points. Degradation of the ligand was delayed in CTNS KD cells in comparison with the control, as expressed after normalization of the signal to the initial value (surface binding at 0 min). (b) The observation was confirmed in control and cystinotic ciPTEC cells (** p<0.01 after 45 min). Treatment with cysteamine resulted in partial rescue of the GST-RAP processing (* p<0.05 after 45 min). Scale bar = 10 μm.

To study endocytosis, cells pre-incubated with GST-RAP ligand were transferred to 37°C to allow internalization of the ligand bound to the cell surface. At indicated time points, cells were fixed and stained for GST-RAP. At earlier time points, the staining was visible in vesicular structures corresponding to endosomes and then gradually disappeared. In CTNS KD HK-2 cells, GST-RAP remained visible for significantly longer time after internalization in comparison to controls (Fig. 4a).

The observation was also confirmed in cystinotic ciPTEC (Fig. 4b). Importantly, pre-treatment of cystinotic ciPTEC with 100 μM cysteamine for 24 h prior to the experiment partially rescued the delayed cargo processing (Fig. 4b).

### Decreased surface expression of megalin is associated with impaired trafficking

**Fig 5:**
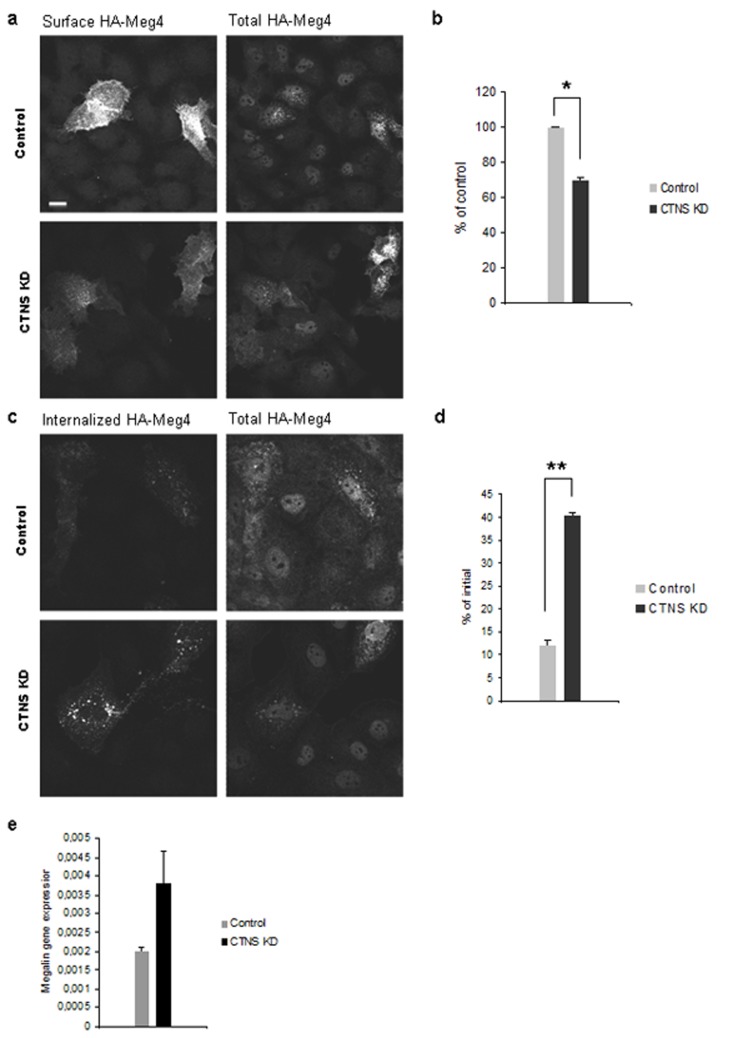
Impaired megalin trafficking in cystinosin-deficient HK-2 cells. (a) Control and CTNS KD HK-2 cells transfected with HA-Meg4 mini-receptor were incubated on ice with anti-HA monoclonal antibodies for 1 h, fixed and stained to visualize the surface HA-Meg4. The surface expression of the protein was decreased in CTNS KD cells in comparison with the control, whereas total amount of HA-Meg4, revealed by staining of permeabilized cells with polyclonal antibodies, remained unchanged. (b) Quantification of fluorescence intensity (2 independent experiments, * p<0.05). (c) Internalization of HA-Meg4 was allowed by moving the cells to 37°C for indicated time points. Images taken after 1 h reveal more HA-Meg4-positive vesicles in CTNS KD cells indicative of deficient recycling of the protein. (d) Measurement of fluorescent intensity after 1 h demonstrated that less HA-Meg4 was internalized in CTNS KD cells at the early time point, but more protein remained visible after 1 h (2 independent experiments, ** p<0.01). (e) qPCR analysis of endogenous megalin expression in control and CTNS KD HK-2 cells, average data from 4 independent experiments.

To exclude the possibility that reduced megalin surface expression was caused by decreased expression of endogenous megalin in cystinosin deficient cells and to study megalin localization and trafficking, we used HA-Meg4 mini-receptor construct transiently expressed in HK-2 cells. This model has been successfully used for this purpose previously [25]. While no major difference in total HA-Meg4 expression was registered between control and CTNS KD cells, the surface expression of HA-Meg4 was decreased by 30% in CTNS KD cells (p<0.05) as assessed by measurement of fluorescence intensity after incubation of cells on ice with anti-HA antibodies (Fig. 5a, b). After transferring the cells to 37°C, HA-Meg4 was internalized to endosomal vesicles. More HA-Meg4 remained visible after 1 h inside CTNS KD cells, indicative of deficient recycling of the receptor from the endosomes (Fig. 5c, d, p<0.01).

We next measured the expression of endogenous megalin in control and CTNS KD HK-2 cells by means of qPCR. The level of megalin expression was not reduced in cystinosin-deficient cells in comparison with the control (Fig. 5e). Together these observations indicate that the reduced surface expression of the receptor and impaired receptor-mediated endocytosis in cystinosin-deficient cells are caused by deficient trafficking of megalin (and, probably, other receptors) rather than decreased gene expression.

### Impaired lysosomal degradation of fluorescently-labelled BSA in cystinotic ciPTEC

**Fig 6:**
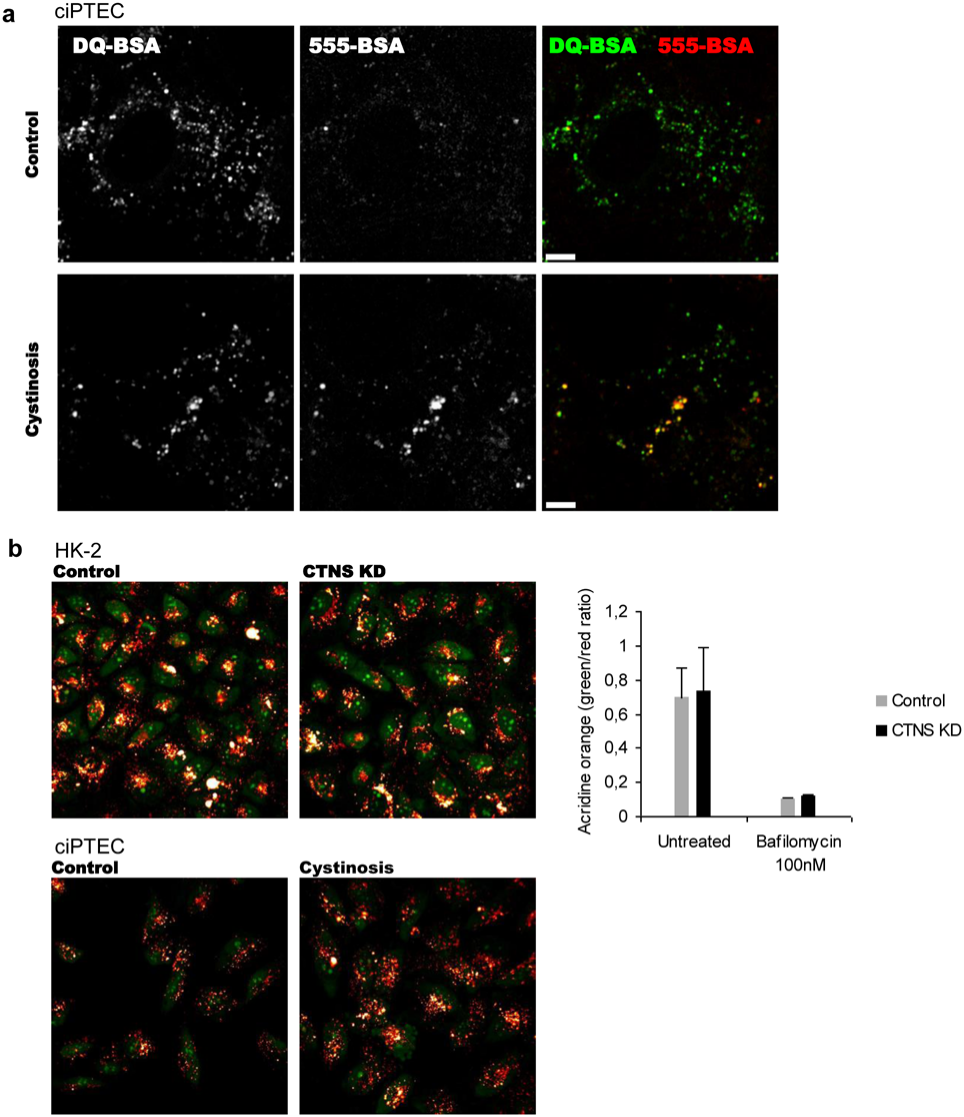
Lysosomal degradation is impaired in cystinosis cells. (a) Trafficking and degradation of endocytic cargo was evaluated by means of fluorescently labelled Alexa-555-BSA and fluorogenic substrate DQ-BSA: control and cystinotic ciPTEC cells were incubated with 5 μg/ml of each substrate for 2 h, washed and analyzed under confocal microscope (live imaging). Quantification of fluorescence intensity showed no decrease in DQ-BSA degradation in cystinotic cells. Incubation of cells with 5 μg/ml of fluorescently labelled 555-BSA for 2 h resulted in a prominent accumulation of the substrate in cystinotic cells in comparison with the control. Scale bar = 10 μm. (b) Staining with acidophilic dye acridine orange (AO) demonstrated normal acidification of lysosomes in cystinosin-deficient cells. Quantification of lysosomal pH by the acridine orange green/red fluorescence intensity revealed no difference between cystinosin-deficient and control cells.

To assess the endocytosis and processing of cargo, exclusively destined for lysosomal degradation, we performed a pulse-chase experiment in ciPTEC cells using fluorescently-labelled BSA as a ligand. We used Alexa-555-labelled BSA to trace the internalization and trafficking together with DQ-BSA green to visualize the lysosomal degradation sites. As expected, 555-BSA processing was delayed in cystinosis cells in comparison with the control, leading to a prominent accumulation after 2 hours. Strong DQ-BSA green fluorescence was observed in the same compartments where 555-BSA accumulation took place, identifying them as lysosomes (Fig. 6a). At the same time, staining with acridine orange dye demonstrated that acidification of lysosomes and lysosome permeability were not impaired in cystinosin-deficient cells (Fig. 6b) [34].

Together these results indicate delayed processing of endocytic cargo and suggest that cystinosin deficiency affects the surface pool of multiligand receptors that can explain reduced protein reabsorption.

## Discussion

In this paper we describe a detailed study of receptor-mediated endocytosis in two models of human proximal tubular cells deficient for cystinosin and demonstrate an abnormal morphology of endosomal and lysosomal compartments and defective endocytosis.

We used proximal tubular cells from adult kidney (HK-2 cells), in which cystinosin was down-regulated by cystinosin-specific siRNA, and conditionally immortalized proximal tubular cells derived from urine of a cystinosis patient bearing a homozygous deletion of the *CTNS* gene. Although using immortalized cell lines has several disadvantages, such as lower expression levels of proximal tubular receptors and transporters and difficulties to study cell proliferation rates, these models offered a possibility to explore the molecular events induced by the acute or chronic loss of expression of cystinosin in human cells. Indeed, the acute down-regulation of cystinosin induced in HK2 cells with specific siRNA allowed us to explore direct consequences of cystinosin deficiency limiting, on the one side, the effects related to inter-individual variability, which is intrinsic to the study of patients-derived (or animal model-derived) cells [19], and, on the other side, effects linked to adaptation phenomena to cystinosin deficiency, which are likely to be less developed in CTNS KD cells compared to cells derived from patients or from Ctns^-/-^ mice tissue. Furthermore, by comparing the two cystinosin deficiency cell models (acute KD vs cells from patients) we had an opportunity to discriminate between direct and indirect consequences induced by the loss of function of the protein. We found that cystinosin down-regulation leads to an acute disorganization of the endo-lysosomal compartments characterized by the decreased expression of the endocytic receptors on the cell surface, the delayed processing of the endocytic ligands, and the clustering of early and late endosomes and lysosomes in the perinuclear region. Only some of these endocytic dysfunctions could be rescued by cystine-lowering agent cysteamine. It therefore likely that cystinosin, next to its cystine transporting activity, plays a role in cellular vesicle trafficking.

Being aware of the intrinsic limitations of using cell models to study complex pathogenic processes, we were reassured in verifying that many of our findings in human cystinotic cells are in concordance with those recently published in a mouse model of cystinosis [20, 21]. The most important observation is the reduced surface expression of multi-ligand receptors megalin and other receptors and transporters demonstrated in kidney tissue of the Ctns^-/-^ mice [20, 21].

The surface expression of multiligand receptors is strictly dependent on their level of expression and on their correct trafficking from the ER and Golgi apparatus and on the recycling processes [35]. During endocytosis, ligand-bound surface receptors are internalized into early and then late endosomes where highly organized sorting processes take place. Molecules bound for fast recycling to the cell surface are sorted into the recycling vesicles from the early endosomal compartment. Slow recycling is going through early and late endosomes via a specialized Rab-11-positive perinuclear compartment [35].

The reduced surface expression of these receptors has been reported by Raggi et al. as a consequence of the dedifferentiation of proximal tubular cells from Ctns^-/-^ mice, a process associated with an activation of the transcription factor ZONAB (zonula occludens (ZO-1)-associated nucleic binding protein), which promotes cell proliferation and directly inhibits the expression of megalin and cubilin. However, it remained uncertain whether the activation of proliferation program is an initial pathogenic event triggered by the absence of cystinosin or a compensatory mechanism aiming to replenish cell losses. In our study we report that the acute down-regulation of the *CTNS* gene primarily reduced surface expression of megalin without affecting the total levels of this protein, thus suggesting a role of cystinosin in directing megalin trafficking.

Interestingly, the reduced expression on the cell surface could be partially rescued already after 24 hours of cysteamine treatment in cystinotic ciPTEC cells that were shown to accumulate significant amounts of cystine under normal culture conditions [19]. In addition to mistrafficking at the level of the early-recycling endosomal compartments we found that cystinosin depletion induced alterations in the late endosomal/lysosomal compartments. These include a delayed degradation of lysosomal substrates and a mislocalization of late endosomes/lysosomes.

The impaired degradation capacity of lysosomes had previously been reported both in patients with cystinosis [36] and in animal models, although no overt evidence of lysosomal enzyme deficiency has been demonstrated [36, 20]. Interestingly, cysteamine almost completely restored delayed cargo processing pointing to the important role of the lysosomal cystine storage in this cellular defect. The impaired degradation capacity of the lysosomes was accompanied by the mislocalization of these organelles that appear clustered in the perinuclear area. Looking for the causes underlying this phenomenon we investigated the impact of the *CTNS* knock-down on motors and their regulators involved in late endosomal and lysosomal motility. Lysosomes undergo bidirectional trafficking along microtubules driven by minus-end and plus-end directed motors [37]. As the motor protein kinesin-1 mediates the plus-end (i.e. periphery) directed motility, we investigated whether an impaired association of kinesin-1 with lysosomes could be responsible for their perinuclear clustering. In fact we found that kinesin-1 was even more associated to lysosomes in cystinosin-depleted cells as compared to control cells, thus discarding the possibility that the cystinosis defect impairs the binding of plus-end directed motor to its cargoes. Interestingly, the potential involvement of kinesins in the pathogenesis of proximal tubular dysfunction has recently been demonstrated in Dent’s disease (another condition presenting with renal Fanconi syndrome) via a direct interaction between ClC-5 chloride-proton exchanger and another kinesin family member kinesin-2 (KIF3B) [38].

Different alternative possibilities can be considered. Firstly, the perinuclear clustering of lysosomes may be due to the inability to anchor them at the cell periphery. This anchoring is achieved by at least two different means: one involving the switch of lysosomes and of lysosome-related organelles from microtubule tracks to peripheral microfilament meshwork [37] and the other involving the cholesterol-regulated contact sites between lysosomes and the ER involving ORP1L [39]. The microtubule-actin meshwork switch is driven by the small GTPase Rab27 and the actin-based motor myosin Va [40, 41]. Interestingly, we found that cystinosin depletion reduced the mRNA levels for Rab27 by 40% (data not shown), and Johnson et al. found that the endocytic defects can be partially corrected by overexpression of Rab27 in cells from Ctns^-/-^ mice that also show lower Rab27 levels as compared to control [42]. These results suggest that a defective peripheral anchoring may underlie the perinuclear clustering of lysosomes in cystinosin-deficient cells. Alternatively, a possible alteration in cholesterol content of cystinosin-depleted lysosomes might impair the ORP1-mediated peripheral anchoring of lysosome to the ER.

The relationships existing between the functional impairment and the altered positioning of lysosomes in cystinosis remain an important subject for future studies.

## Supporting Information

S1 Fig Depletion of cystine accumulation in cystinosis ciPTEC cells in course of prolonged treatment with cysteamine. ciPTEC cells were incubated for indicated time points with 100 μM of cysteamine. Cysteamine-containing culture medium was refreshed daily. Cystine content of each sample was normalized by protein concentration and expressed as % of the initial. The graph represents averaged data from 2 different cystinosis ciPTEC lines deriving from patients bearing a homozygous 57 kb deletion of the *CTNS* gene.
(TIF)
