# Supplementary figures and images for: Endo-Lysosomal Dysfunction in Human Proximal Tubular Epithelial Cells Deficient for Lysosomal Cystine Transporter Cystinosin
Source: PLoS One. 2015 Mar 26;10(3):e0120998. doi: 10.1371/journal.pone.0120998 (PMC4374958; doi:10.1371/journal.pone.0120998)

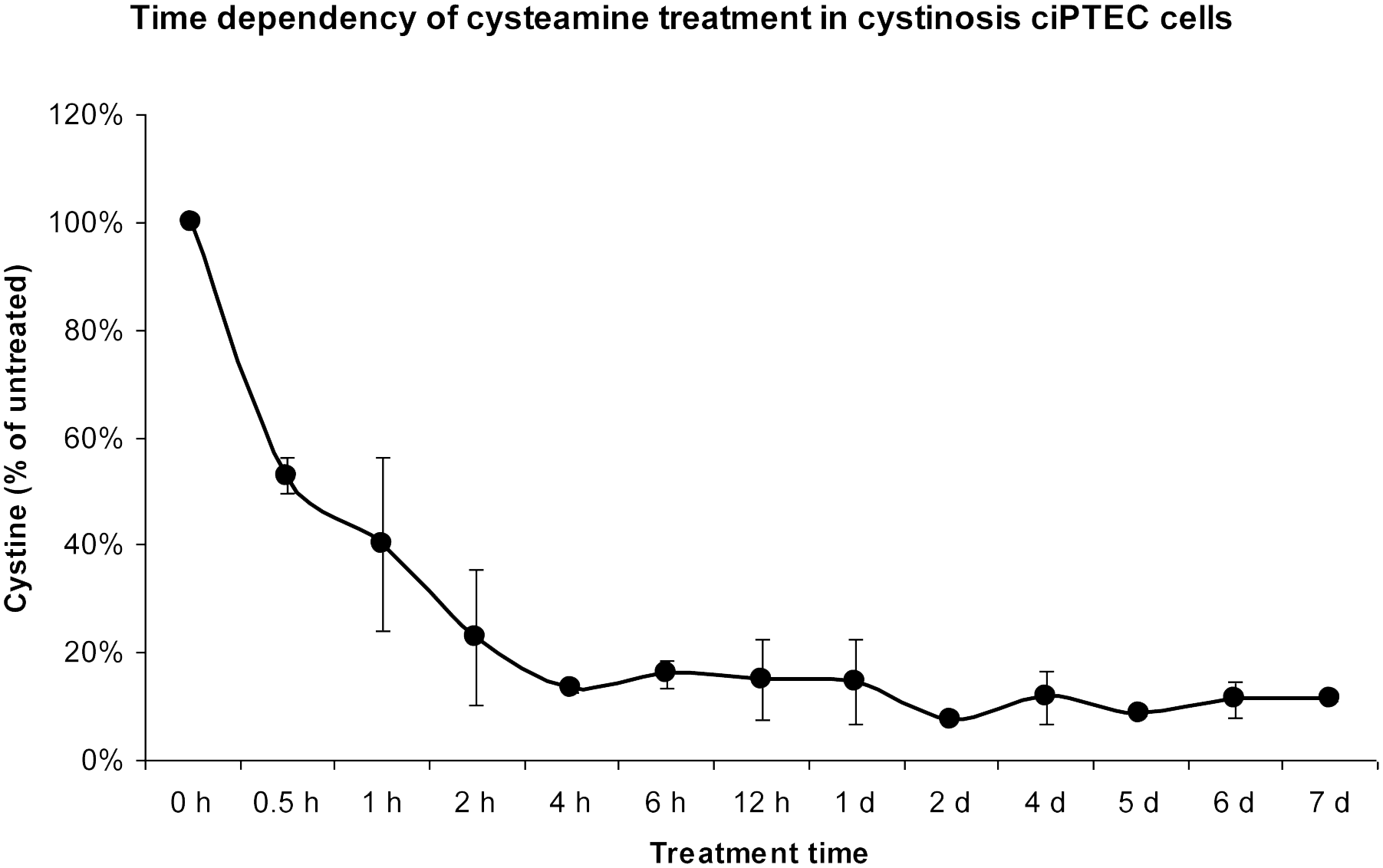

Supplement: S1 Fig — ciPTEC cells were incubated for indicated time points with 100 μM of cysteamine. Cysteamine-containing culture medium was refreshed daily. Cystine content of each sample was normalized by protein concentration and expressed as % of the initial. The graph represents averaged data from 2 different cystinosis ciPTEC lines deriving from patients bearing a homozygous 57 kb deletion of the CTNS gene. (TIF) [file pone.0120998.s001.tif]
